# Bibliography

**Published:** 1886-11

**Authors:** 


### BIBLIOGRAPHY.

DISEASES OF THE NERVES, MUSCLES AND SKIN, being Vol. III. of Dr. Hermann Eichhorst's Handbook of Practical Medicine, and Vol. X. of Wood's Library of Standard Medical Authors, 1886, (consisting of 12 vols., price $15.00.) Sold only by subscription. William Wood & Co., New York. The author takes right hold of his subject, and disposes of it *secundem artem* in a most thorough and scientific manner. This volume begins where volume two left off, at section V, Diseases of the Nerves, which are classified and treated seriatim, beginning at peripheral nerves—motor and sensory; then of the spinal cord, and finally of the brain. Section VI. treats of diseases of the skin upon a classification of six divisions: 1st—Dermatidides, under which head he describes: A, Erythematous inflammation, such as urticaria. 2—Erythema nodosum. 3—Erythema exudativum multiform, [a pretty sweeping classification.] 4—Acrodynia. 5—Pellagra, B, vesicle-like inflammations: 1, Eczema; 2, milearia, etc., etc.

This is about as good and convenient a classification as we have seen.

---

ELECTROLYSIS, its Theoretical Consideration and its Therapeutical and Surgical Applications. By Robert Amory, A. M., M. D., Member of the Massachusetts Medical Society; Fellow of the American Academy of Arts and Sciences; Fellow of the American Academy of Medicine, etc., etc. Octavo, 314 pages. Illustrated by nearly one hundred fine wood engravings. Supplied only to subscribers for "Wood's Library of Standard Medical Authors," for 1886 (12 vols., price, $15.00) of which this is Vol. VIII. New York, William Wood & Company.

This is the August number of this popular series. It is written in a singularly clear and easily comprehended style for a book of such a technical character. Beginning at the beginning of the subject, it carries the reader through Physics in Electrolysis. The kind of batteries necessary for Electrolysis. The resistance and diffusion of the Electrical current, the effects of electricity upon the human body. Destruction of Living Tissue by Electrolysis, Modes of Employing Electrolysis, Application of ditto, to treatment of disease in general, and to certain diseases in particular, such as exophthalmic Goitre and Hypertrichosis. Then a chapter on the measurement of currents of Electricity is given. The book closes with a description of apparatus and instruments used in treatment by electrolysis of the living tissue, and a general summary, in which are discussed some of the causes of the resolutive action from galvanism of the tissues by surface application of the electrodes, or from their electro-puncture.

Unlike certain pedantic writers, we could name, this author does not presuppose a thorough knowledge of the subject on the part of the reader; but he takes pains to make details clear, even in the rudiments of the subject. It is a good and unique little book.

---

A MANUAL OF ANIMAL VACCINATION, PRECEDED BY CONSIDERATIONS ON VACCINATION IN GENERAL. By Dr. E. Warlomotn, Member of the Royal Academy of Medicine, of Belgium;

Founder of the State Vaccine Institute of Belgium; Director of the "Institut Vaccinal de Belgique," at Brussels, etc. Translated and edited by Arthur J. Harris, M. D., Assistant Physician and Joint Lecturer to St. John's Hospital for Diseases of the Skin; Consulting Physician to the Association for the Supply of Pure Vaccine Lymph; Honorary Secretary to the Willan Society of London; Treasurer to the Sloane Society; Late Honorary Medical Officer to the Holloway and North Islington Dispensary, etc.. etc. With an appendix, showing the results of re-vaccination, and the comparative utility of animal vaccine. Philadelphia: John Wyeth & Brother, 1412, 1414 and 1416 Walnut Street.

This is, we believe, the most exhaustive work on the subject recently published, and as Dr. Warlomont is the founder of the State Vaccine Institute of Belgium, and for a number of years was the director, his experience entitles his opinion and advice to consideration as an authority on vaccination, and everything connected with bovine lymph. It is a book which will prove of much interest to the medical profession, and contains information in detail that will be a benefit also to sanitary committees, boards of health, etc., etc. Published by John Wyeth & Brother, who are *also* authority on vaccine virus.

